# Homology Analysis of *Polistes dominula* and *Vespula* spp. Venoms: A Comparative In Vitro and In Silico Study

**DOI:** 10.3390/toxins18040190

**Published:** 2026-04-18

**Authors:** María Morales, Alicia Jordá Marín, Bárbara Cases, Louise Wallace, Dolores Hernández Fernández De Rojas

**Affiliations:** 1 Allergy Therapeutics Ibérica S.L.U., 28805 Madrid, Spain; alicia.jorda@allergytherapeutics.com (A.J.M.); barbara.cases@allergytherapeutics.com (B.C.); dolores.hernandez@allergytherapeutics.com (D.H.F.D.R.); 2 Allergy Therapeutics (UK) Limited, Worthing BN14 8SA, UK; louise.wallace@allergytherapeutics.com

**Keywords:** allergen homologous groups, *Polistes dominula*, venom immunotherapy, vespids, *Vespula* spp.

## Abstract

A homologous classification for vespid venoms is missing. This study compared *Polistes dominula* and *Vespula* spp. venoms to evaluate their homology level. *P. dominula* and *Vespula* spp. extracts, including *V. germanica*, *V. maculifrons*, *V. pensylvanica*, *V. alascensis*, and *V. squamosa* in equal proportions, were generated from venom sacs and were subjected to sodium dodecyl sulfate–polyacrylamide gel electrophoresis (SDS-PAGE) and Western blot using *Vespula*-positive sera. Bands described as allergenic were excised and sequenced through Liquid Chromatography–Mass Spectrometry tandem analysis (LC-MS/MS) to confirm their identity. Phospholipase (group 1) and hyaluronidase (group 2) enzymatic activities were measured. Group 1 and 5 3-D structures and sequence identity were analyzed in silico. The results showed that the *P. dominula* and *Vespula* spp. venom extracts exhibit similar protein profiles and comparable allergen composition, with phospholipase and hyaluronidase activities. The structures of Pol d 1 and Ves v 1 and Pol d 5 and Ves v 5 were highly similar, and the identity levels were high across and within the *Polistes* and *Vespula* genera (≥50%). These results suggest the inclusion of venoms from *Polistes* and *Vespula* genera as candidates to create a new homologous group for wasp venoms and indicate that the currently described homologous groups require revision.

## 1. Introduction

Allergy to Hymenoptera venom, including honeybees, vespids, and ants, may cause potentially life-threatening systemic reactions, with bee and wasp stings being the most frequent causes [1,2]. In Europe, the reported rates of systemic sting reactions range from 0.3% to 7.5% in adults and from 0.15% to 0.8% in children [3]. The most common vespids (i.e., *Vespidae* family) in Europe are the *Vespula* spp., which belong to the Vespinae subfamily, and *Polistes dominula*, which belongs to the Polistinae subfamily and is distributed across the continent [4,5]. *P. dominula* is present at lower densities, particularly in Northern Europe, but it is most common in Mediterranean regions and is increasingly expanding due to climate change [6]. Venom immunotherapy (VIT) with venom extracts is recommended in individuals with a history of systemic reactions to Hymenoptera stings, with reported effectiveness rates between 91% and 99% for vespid venom and between 77% and 84% for bee venom [7,8].

In Europe, the Committee for Medicinal Products for Human Use (CHMP) is responsible for the human medicines of the European Medicines Agency (EMA) (European Medicines Agency). The CHMP regulates allergen extracts, allergen immunotherapy and diagnostic products, among others. The CHMP guideline on allergen products states that allergen extracts and products may be grouped into homologous groups, allowing the extrapolation of some data across products with identical formulations and preparation from the same groups [9].

According to the information available, the principle for generating homologous allergen groups was based on similarities regarding the biochemical composition and homology/cross-reactivity of allergens [10]. This review established homologous groups among mites and pollen from trees, grasses, and weeds, but the allergen sources for insect venoms and other allergens from animal sources were not included in any group. However, vespid venoms are suggested as candidates for another homologous group [10]. Since the publication of the guidelines in 2007, a vast amount of information regarding Hymenoptera venom allergens has been generated, and several new allergens have been identified and sequenced [11,12,13]. Current evidence has shown that venoms from *Vespula* and *Polistes* genera contain allergens from the same biochemical groups (i.e., protein families) [11,12,13]. The five allergens identified in *P. dominula*, including phospholipase A1 (Pol d 1), hyaluronidase (Pol d 2), dipeptidyl peptidase IV (Pol d 3), serine protease (Pol d 4), and antigen 5 (Pol d 5), have also been identified in *Vespula* spp., except for group 4, with a group 6 allergen additionally identified in *V. vulgaris* (Ves v 6) [12,14].

Knowledge of allergen composition and homologies among allergen sources is key for improving patient diagnosis based on component-resolved diagnosis. Given the lack of homologous classification for vespid venoms, including those from the most common species in Europe, a comparison is needed to establish a classification into homologous groups. This study compared venoms from *P. dominula* and *Vespula* spp. using state-of-the-art methods to evaluate their homology level.

## 2. Results

### 2.1. Protein and Allergenic Profiles of P. dominula and Vespula spp. Venoms

The first step of this comparison study was the analysis of protein extracts of the venom sacs obtained from *P. dominula* and a mix of five *Vespula* species using SDS-PAGE and Western blot with *Vespula*-specific positive sera to compare their protein and allergenicity profiles, respectively.

**Figure 1:**
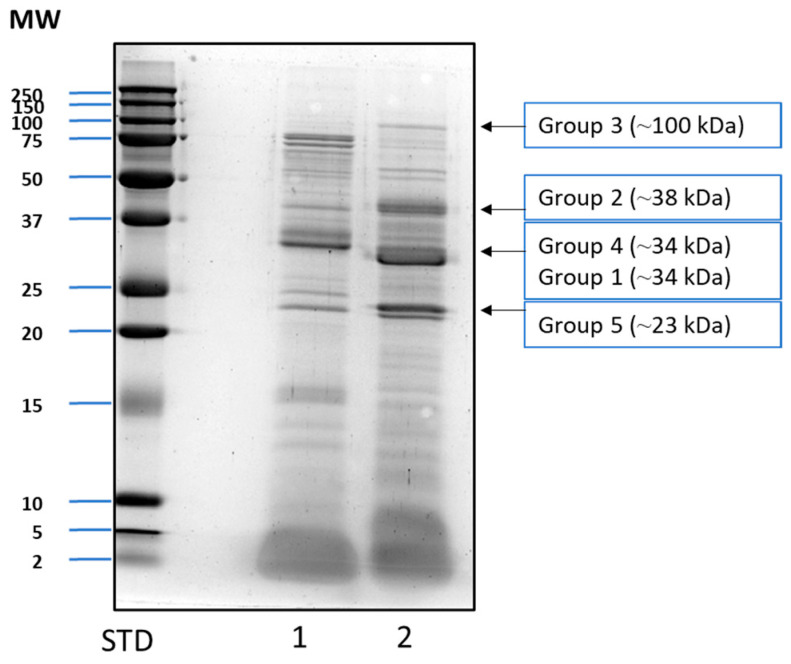
SDS-PAGE followed by Coomassie blue staining of venom extracts from *Polistes dominula* (lane 1) and a mix of five *Vespula* species (lane 2). The standard (STD) corresponds to the Precision Plus Dual Standard marker (BioRad) and shows the predicted molecular weight (MW) in kDa. The approximate molecular weights corresponding to the various groups of wasp allergens are labeled and indicated with arrows.

SDS-PAGE followed by Coomassie blue staining revealed that the venoms from *P. dominula* and a mix of five *Vespula* species displayed similar, though not identical, protein profiles, where the main allergen bands could be clearly identified at their expected approximate molecular weights (Figure 1).

**Figure 2:**
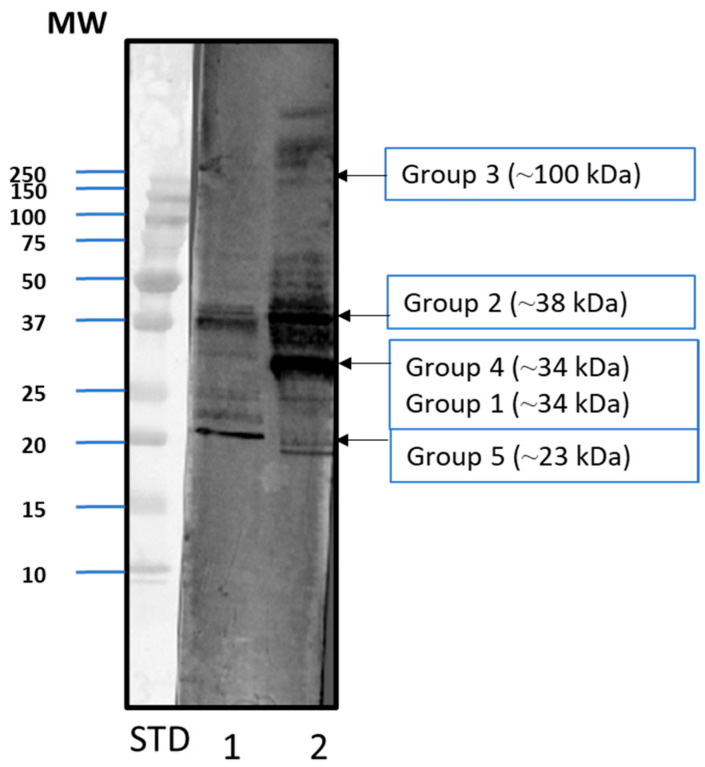
Western blot of venom extracts from *Polistes dominula* (lane 1) and a mix of five *Vespula* species (lane 2) using commercial *Vespula*-specific positive sera pool. The standard (STD) corresponds to the Kaleidoscope marker (Bio-Rad), and the approximate molecular weight (MW) is given in kDa. The approximate molecular weights corresponding to the various groups of wasp allergens are labeled and indicated with arrows.

To determine whether the bands corresponded to actual allergens and compare the allergenic protein profiles, protein extracts from both venoms were analyzed through Western blot using a pool of *Vespula*-specific positive sera. Both extracts showed bands corresponding to groups 1, 2, 4 and 5 (Figure 2). However, a different recognition pattern was observed between both Hymenoptera genera. The signal was weaker with *Polistes* than with *Vespula* as the pool was selected according to *Vespula* sIgE. The Pol d 3 band was not clearly observed due to its lower apparent abundance in the extract, as shown by Coomassie blue staining, which revealed a low intensity band at the expected size in *P. dominula* venom extract (Figure 1).

To confirm the identity of the protein bands, Liquid Chromatography–Mass Spectrometry tandem analysis (LC-MS/MS) was performed on the excised bands corresponding to the molecular weights of the described allergens (approx. 100, 38, 33/34 and 23 kDa) (see Figure 1). The analysis confirmed that these bands correspond to allergen groups 3, 1, 4 and 5, respectively, of *P. dominula*, and 3, 2, 1 and 5, respectively, of *Vespula* spp. (Table 1). Group 3 allergens were present in lower abundance but were nevertheless identified in both extracts. Group 2 allergens were only identified for *Vespula* spp., while Pol d 2 was not identified (possibly due to its low abundance in the source). Group 4 was not identified in *Vespula* spp. and was identified only in *P. dominula* (Table 1).

### 2.2. Phospholipase and Hyaluronidase Activities of Vespula spp. and P. dominula Venoms

**Figure 3:**
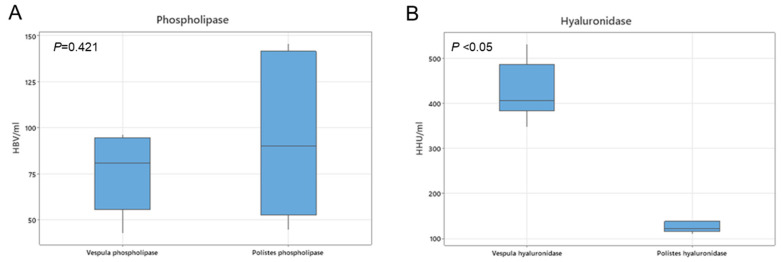
Quantification of the enzyme activities for (**A**) phospholipase (HBV/mL) and (**B**) hyaluronidase (HHU/mL). The box represents the interquartile range, the whiskers represent the minimum and maximum, and the horizontal line inside the box represents the median obtained from five batches of *P. dominula* venom extract and six of *Vespula* spp. venom extracts. *p*-values for comparisons between genera were calculated using a *t*-test.

The allergenic group 1 and group 2 proteins correspond to phospholipase and hyaluronidase, respectively, whose activities can be detected and quantified using enzymatic assays. Activity assays for the presence of both phospholipase and hyaluronidase on venom extracts were used to characterize and compare the *Vespula* spp. and *P. dominula* venoms. The results show that both the phospholipase A1 (group 1) and hyaluronidase (group 2) activities were measured in the *Vespula* spp. and *P. dominula* venom extracts (Figure 3), despite Pol d 2 (hyaluronidase) not being identified in the LC-MS/MS analysis. The levels of phospholipase activity measured were similar for both venom extracts (Figure 3A), whereas hyaluronidase activity was higher in the five-*Vespula* venom mix than in *P. dominula* venom (Figure 3B), consistent with the LC-MS/MS results.

### 2.3. Sequence Identity of Group 1, Group 5, Group 2, and Group 3 Allergens from Polistes spp. and Vespula spp. Venoms

While these experiments allowed us to confirm that the previously identified wasp allergens are present in *P. dominula* and *Vespula* spp. venoms, the percentage similarities among allergens from the same group within *Vespula* and *Polistes* genera remain unknown. Therefore, sequence identity was analyzed in silico using Clustal Omega multiple sequence alignment program (EMBL’s European Bioinformatics Institute, version 1.2.4), focusing on the group 1 and group 5 allergens, which are considered major allergens and distinct markers of wasp sensitization [15]. Group 2 and group 3 allergens, described in both genera, were also compared. The corresponding sequences described in the databases for all *Polistes* and *Vespula* species available were obtained, namely *Polistes annularis* (Pol a 1 and Pol a 5), *P. dominula* (Pol d 1, Pol d 5, Pol d 2 and Pol d3), *Polistes gallicus* (Pol g 5), *Polistes fuscatus* (Pol f 5), and *Polistes exclamans* (Pol e 5) and *V. germanica* (Ves g 1, Ves g 5, and Ves g 2), *Vespula flavopilosa* (Ves f 5), *V. maculifrons* (Ves m 1 and Ves m 5), *V. pensylvanica* (Ves p 5), *V. vulgaris* (Ves v 1, Ves v 5, Ves v 2, and Ves v3), *V. squamosa* (Ves s 1 and Ves s 5), and *Vespula vidua* (Ves vi 5).

**Figure 4:**
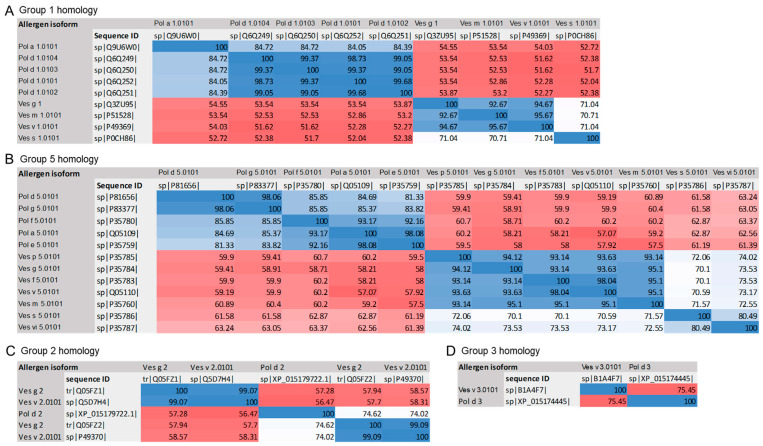
Identities (%) between group 1 (**A**), group 5 (**B**) (major allergens), group 2 (**C**), and group 3 (**D**) isoform sequences of different *Polistes* (*P. dominula*, *P. gallicus*, *P. fuscatus*, *P. annularis*, and *P. exclamans*) and *Vespula* (*V. germanica*, *V. pensylvanica*, *V. flavopilosa*, *V. maculifrons*, *V. vulgaris*, *V. squamosa* and *V. vidua*) species. Group 4 and group 6 were not compared, since group 4 is not described in *Vespula* as an allergen, and group 6 is not described in *Polistes*. The colors correspond to identity levels, ranging from dark red for lowest identities to dark blue for highest identities.

Sequence identities between *Polistes* group 1 allergens (Pol d 1 and Pol a 1) were over 80%. The sequence identity among *Vespula* group 1 allergens was over 90%, except for Ves s 1, which was 70%. The overall identity among both genera was over 50% (Figure 4).

Analysis of group 5 wasp allergen identities yielded similar results. *Polistes* group 5 allergens (Pol d 5, Pol g 5, Pol f 5, Pol a 5, and Pol e 5) shared a sequence identity of over 80%. *Vespula* group 5 allergens shared a sequence identity over 90%, except for Ves s 5 and Ves vi 5, which was 70% (Figure 4).

Regarding minor allergens, sequence identities between genera were >56% for group 2 allergens and >75% for group 3 allergens. *Vespula* group 2 allergens shared a sequence identity over 99%.

For group 5, two sequence subsets displayed the highest identity within each genera: For *Polistes*, the subsets were 1) *P. dominula* and *P. gallicus*, with 98.06% sequence identity, and 2) *P. fuscatus*, *P. annularis* and *P. exclamans*, with 93.17% and 92.17% sequence identity, and, for *Vespula*, the subsets were 1) *V. germanica*, *V. pensylvanica*, *V. flavopilosa*, *V. maculifrons*, and *V. vulgaris*, with sequences identities ranging from 93.14% to 94.12%, and 2) *V. squamosa* and *V. vidua*. Therefore, two differentiated groups of *Vespula* species appear to exist: one comprising *V. germanica*, *V. pensylvanica*, *V. flavopilosa*, *V. maculifrons* and *V. vulgaris*, which are more closely related phylogenetically, and another comprising *V. squamosa* and *V. vidua* (Figure 4).

Comparison between both genera (*Polistes* spp. and *Vespula* spp.) showed sequence identities >50% in multiple sequence alignments of group 1 and 5 protein sequences (Figures S1 and S3).

### 2.4. 3D Structures of Group 1 and Group 5

**Figure 5:**
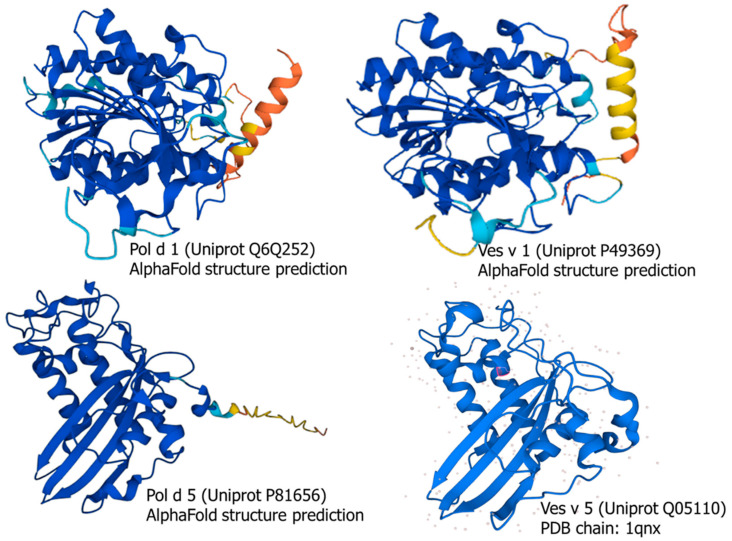
Structural modeling of the indicated sequences retrieved from UniProt (Pol d 1, Ves v 1, and Pol d 5) and Ves v 5 crystallographic structure. Colors indicate the confidence of the structure model (dark blue means very high, clear blue is confident, yellow means low confidence and orange means very low).

To further confirm the similarities within homologous groups, the 3D structures of the major allergens (group 1 and group 5) were analyzed. The structural modeling of Pol d 1, Ves v 1, and Pol d 5, together with the only available crystallographic structure of Ves v 5, revealed similar tertiary structures within each group (Figure 5).

**Figure 6:**
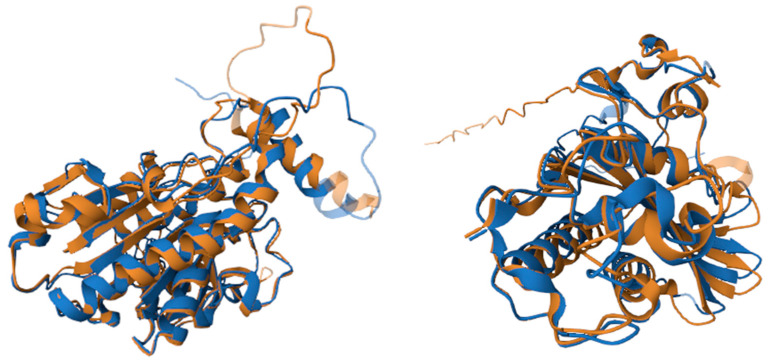
3D alignment of group 1 (Pol d 1.0101 in brown; Ves v 1.0101 in blue) at the left and group 5 (Pol d 5.0101 in brown; Ves v 5.0101 in blue) at the right.

3D alignment between group 1 and 5 allergens was performed (Figure 6). The alignment of group 1 was performed using Uniprot entries Q6Q252 (Pol d 1.0101) and P49369 (Ves v 1.0101). Group 5 3D alignment was performed using Uniprot entries P81656 (Pol d 5.0101) and Q05110 (Ves v 5.0101). The identity for group 1 was 49%, and for group 5 it was 58%. The greatest differences were observed in the amino-terminal of the proteins, which correspond to the region of lower sequence identity (Figures S1 and S3). The differences in alignment of group 1 proteins involve a loop without defined secondary structure and the displacement of the alpha-helix. The difference in alignment of group 5 also corresponds to the loop without a secondary structure. This analysis has the limitation that the amino-terminal sequences are the ones with a lower confidence in structural models.

## 3. Discussion

This study compared extracted venoms from *P. dominula* and a mixture of five *Vespula* species and demonstrated that both extracts exhibit similar protein profiles, and these were identified using LC-MS/MS analysis. The presence of phospholipase and hyaluronidase activities, corresponding to group 1 and group 2 allergens, respectively, was also detected. The in silico structural analyses demonstrated high similarities between Pol d 1 and Ves v 1 and Pol d 5 and Ves v 5. Furthermore, high sequence identities among and within groups 1 and 5 from *Polistes* and *Vespula* genera were also confirmed. The results from this comparative analysis suggest the inclusion of *Polistes* and *Vespula* species in a new allergen homologous group.

The results from this study regarding the identification of allergens using LC-MS/MS and the detection of enzymatic activity for phospholipase and hyaluronidase were consistent with the current knowledge of the allergens present in these species [11,12]. Comprehensive LC-MS/MS analysis revealed that allergens belonging to group 1 (phospholipase) and group 5 (antigen 5), considered major allergens, were present in *P. dominula* and in all *Vespula* species analyzed [11,12], except group 1 from *V. pensylvanica*, as expected, as its sequence is not yet available. These allergens are considered markers of wasp sensitization since they enable discrimination from honeybee sensitization [16].

Groups 1 and 5 are discriminatory between wasp and honeybee venom sensitization. In contrast, these allergens have shown a high cross-reactivity among wasp species, making discrimination difficult between *Vespula* spp. and *P. dominula* venom sensitization [17]. Studies have demonstrated cross-reactivity between *P. dominula* and *V. vulgaris* phospholipases and antigen 5, while other reports assessing multiple vespid species using different methods, including sIgE analysis, inhibition assays, and the basophil activation test, obtained similar results [16,18]. The observed cross-reactivity is likely due to the high homology between allergens and is independent of cross-reactive carbohydrate determinants [19]. In this regard, in silico studies comparing phospholipases from different *Polistes* and *Vespula* species showed a high sequence identity among wasp species [20,21]. Moreover, homology seems to be preserved at the structural level according to 3D modeling studies, suggesting that phospholipase conformational epitopes (i.e., B epitopes) are highly conserved, similar to this study [20,21].

In clinical practice, patients allergic to *Vespula* venom from regions with little or no presence of *Polistes* displayed detectable Pol d 5 sIgE, supporting cross-reactivity [22]. Another study showed that specific IgE levels of Antigen 5 allergens and phospholipases allowed the identification of the primary sensitizer in two-thirds of patients, despite the significant cross-reactivity between *P. dominula* (Pol d 5) and the different *Vespula* species spp. (Ves v 5, Ves g 5, Ves s 5, Ves m 5, and Ves p 5) [16,23]. Unfortunately, for the vespid phospholipases, Pol d 1 remains commercially unavailable for component-resolved diagnosis, limiting the ability to discriminate this species routinely [23].

Regarding minor allergens, hyaluronidase (Pol d 2) was not identified in LC-MS/MS, but its hyaluronidase activity was detected in an enzymatic assay, confirming the presence of this allergen in the extracts. These discrepant results may be due to the low relative abundance of hyaluronidase, which might have precluded its detection by LC-MS/MS, whereas it was detected in the more sensitive enzymatic assay. Hyaluronidase activity was lower in *P. dominula* than in *Vespula* spp. venom, consistent with the mass spectrometry results. Vespid hyaluronidases (Ves v 2/Pol d 2) are not considered major allergens but have shown conserved secondary and tertiary structures that may play a significant role in IgE binding [24]. Positive group 2 sensitization may reflect cross-reactivity with Api m 2, since Ves v 2/Pol d 2 in vitro-positive patients show negative responses in basophil activation tests (BATs) for recombinant Pol d 2 or Ves v 2, while Pol d 2/Ves v 2-positive patients were also positive to Api m 2 [24].

Similarly, Pol d 3 (dipeptidyl peptidase IV) was also identified and apparent in our SDS-PAGE despite its previously reported low abundance (see Figure 1) [25]. Pol d 3 has been proposed as a major allergen in *P. dominula* venom, with a significant cross-reactivity among wasp species due to the presence of conserved IgE epitopes [26]. The identification and comprehensive analysis of these minor allergens in this study add to the available data on wasp venoms and provide valuable information suggesting wasp venoms as an allergen homologous group.

The in silico sequence analysis performed in this study focused on the major allergens (group 1 and group 5), since they are the most relevant. Their sequence identity was over 50% between genera, and higher within genera. We compared the tertiary structures of the major allergens (Ves v 1 vs. Pol d 1 and Ves v 5 vs. Pol d 5) and found comparable structures, suggesting similar allergenicity despite lower sequence identities (>50%). This is consistent with a previous study comparing Pol d 5 and Ves v 5 that revealed conserved tertiary structures [23]. In line with these observations, Grosh et al. compared Ves v 2 and Pol d 2 and found high similarities of their 3D tertiary structures despite the lower sequence identities, indicating that the conservation of secondary and tertiary structure may enable cross-reactivity, even when sequence homology is limited [24]. While previous studies have reported sequence identities among *Vespula* allergens, they were limited to some allergens and specific *Vespula* species [27]. In our study, we performed comprehensive sequence analyses including all the identified allergens from five *Vespula* species, adding valuable information regarding the allergen composition in these venoms. Overall, these results suggest a homology between *P. dominula* and *Vespula* spp. venoms.

Homologous allergen groups emerged as a substitute for taxonomic families as the same protein families were identified in allergen sources from different taxonomic families [10]. The homologous groups were established based on the evaluation of the existing literature regarding cross-reactivity and homology [10]. At that time, the identified allergens were Pol d 1, Pol d 4, and Pol d 5 in *P. dominula*, and group 1, group 2, and group 5 allergens in *Vespula* spp. [10]. However, even though the review acknowledged that vespids of the genera *Vespula*, *Dolichovespula*, *Polistes*, and *Vespa* may be good candidates to form a group, the homology among these species was limited to the available data on group 1 and group 5 allergens and was therefore considered insufficient [10]. Since this review, Pol d 2 (hyaluronidase), Pol d 3 (serine protease), and group 3 allergens in *Vespula* (Ves v 3) have been identified [26,28], raising the need to reassess the classification of their allergen sources (i.e., venoms) into a homologous group using state-of-the-art approaches. In this regard, MS allows the identification of individual proteins from complex mixes, as analyzed in this study, providing compelling data supporting the presence of the same allergens in *P. dominula* and *Vespula* spp. venoms.

From the regulatory perspective, homologous allergens should show comparable biological and physicochemical properties of the source material, comparable allergen cross-reactivity or structural homology, an identical formulation of the finished product, and an identical production process for the allergen extract and the finished product [9]. In this regard, the established classification was considered flexible, and studies assessing other allergen sources have proposed the inclusion of other pollens into the established homologous groups [10,29]. In this study, the allergens were obtained from sources with comparable physicochemical and biological properties (i.e., vespid venom sac), following the same procedure, and showed similar allergenic profiles. However, the generalizability of these findings across manufacturers may depend on differences in the production and purification processes used for venom extracts.

Given the lack of a homologous group for Hymenoptera venoms, the results from this study, including the biochemical data from *P. dominula* and five *Vespula* species and in silico data from the *Polistes* and *Vespula* genera, support the potential inclusion of these genera in one homologous group. *Vespula* spp. is distributed across Europe, and the paper wasp *P. dominula*, initially present in Mediterranean regions and Northern Africa, is expanding into Northern Europe, indicating an increasing co-existence of the two species. *Vespula* spp. and *Polistes* spp. genera are widely present in Spain, with a predominance of *Vespula* spp. in the north and of *Polistes* spp. in the center and south of Spain, even though *Polistes* spp. have increased in both areas [30]. Accordingly, patients with a double sensitization to paper wasp and *Vespula* spp. are frequent in Southern Europe and may become more common in Northern areas [27]. Consequently, distinguishing between *P. dominula* and *Vespula* spp. venom allergy after a sting is becoming difficult in more European regions. Identifying the relevant allergen in patients with double sensitization is challenging due to the co-existence of wasp species and cross-reactivity between venoms, but it remains fundamental to ensure appropriate VIT prescription and VIT success [27,31]. In this regard, while this study supports the potential inclusion of *Polistes* spp. and *Vespula* spp. in the same new homologous group, the choice of VIT should nevertheless be species-specific. Clinical evidence shows that, despite the high degree of cross-reactivity between vespid venoms, effective immunotherapy requires the use of the specific venom of the culprit species [31].

The results from this study should be read in the context of the limitations associated with the parameters analyzed, including the lack of experimental assessment of functional cross-reactivity between genera. However, the high conservation of the allergens reported in this study suggests the potential for cross-reactivity between genera. Furthermore, the cross-reactivity of the major allergens (group 1 and group 5) has been extensively demonstrated, and the potential cross-reactivity for group 2 and group 3 allergens has been recently reported [10,24,26,27]. Additionally, *Vespula* spp. venom extract consisted of a mixture of five species, which may mask potential interspecies variability in allergen composition and enzymatic activity. This aspect will be addressed in future studies evaluating individual species. Moreover, venoms from other wasp genera, including *Vespa* and *Dolichovespula*, were not analyzed. Further studies including other *Polistes* species in addition to *Vespa* spp. and *Dolichovespula* spp. are needed to evaluate whether the four social vespid genera may be included in the same homologous group. Finally, this study focused on the composition, sequence, and structural characteristics of venom extracts and did not consider clinical data or functional cross-reactivity, which was beyond its scope. Despite these limitations, this study provided valuable data supporting a potential new wasp venom homologous group, including *P. dominula* and *Vespula* spp. venoms. Future studies addressing functional cross-reactivity will be necessary to confirm this classification.

## 4. Conclusions

Venoms from *P. dominula* and five *Vespula* species showed a comparable allergen composition, sequence homology, and structural conservation. These results suggest that these venoms may represent candidates to create a new homologous group for wasp venoms and indicate that the currently described homologous groups require re-evaluation.

## 5. Materials and Methods

### 5.1. Venom Extraction and Sample Preparation

Venom sacs from *Vespula* spp. and *P. dominula* were extracted using standardized internal protocols (Allergy Therapeutics PLC). Five *Vespula* species were included in the same *Vespula* spp. extract at the same proportions (*V. germanica*, *V. maculifrons*, *V. pensylvanica*, *V. alascensis*, and *V. squamosa*). Briefly, venom glands were obtained through dissection from frozen wasps, and those from the five Vespula species were mixed before venom extraction. The material was homogenized using an appropriate buffer, sterile-filtered, and freeze-dried.

### 5.2. Protein Profile Analysis

The protein profile was determined through sodium dodecyl sulfate–polyacrylamide gel electrophoresis (SDS-PAGE) under reducing conditions in a gradient gel. Venoms from *Vespula* spp. and *Polistes dominula* were reconstituted in saline solution. A total of 10 µg of protein was denatured and reduced using β-mercaptoethanol at 100 °C for 5 min and was loaded onto an AnyKD gel (Bio-Rad Laboratories, Hercules, CA, USA) and stained with Coomassie blue R-250 (Bio-Rad). A molecular marker was also included in the gel.

### 5.3. Allergenic Profile Analysis

The allergenic profile was determined through Western blot. After SDS-PAGE (see previous section), proteins were transferred onto a nitrocellulose membrane, which was incubated overnight at 4 °C with a specific pool of *Vespula*-positive sera (Plasmalab, Everett, WA, USA). Western blots were developed after incubation with anti-human-IgE labeled with peroxidase produced in goat (Sigma-Adrich, St. Louis, MO, USA) and a chemiluminescent reaction with luminol (Revvity, Inc., Waltham, MA, USA) as a substrate.

### 5.4. Quantification of Phospholipase and Hyaluronidase Activities

Phospholipase (group 1) and hyaluronidase (group 2) enzymatic activities were measured in five batches of *P. dominula* venom extract and six of *Vespula* spp. venom extract.

Enzymatic activities were measured through the catalysis of the substrates using egg lecithin or hyaluronic acid for phospholipase or hyaluronidase activities, respectively, in an agarose gel at 37 °C using an internal standard for the calibration curve. Activities were expressed as honeybee venom units (HBV)/mL for phospholipase and Hymenoptera activity (HHU)/mL of hyaluronidase and were compared using a *t*-test.

### 5.5. Protein Sequencing

*P. dominula* and *Vespula* spp. extracts were subjected to SDS-PAGE, where several bands, corresponding to the molecular weight of the described allergens, were excised for sequencing analysis using LC-MS/MS. The selected bands corresponded to the approximate molecular weights of 23, 33 and 100 kDa for *Polistes*, and 23, 34, 38 and 100 kDa for *Vespula*.

Sequencing analyses were performed using the Plataforma de Proteómica Service (Parc Cientific de Barcelona, Universitat de Barcelona). Excised gel bands were washed with 50 mM ammonium bicarbonate and acetonitrile, were reduced with 20 mM DTT (30 min at 60 °C) and were subsequently alkylated with 55 mM iodoacetamide (30 min at 25 °C in the dark). Proteins were digested in-gel using sequencing grade modified trypsin (Promega, Madison, WI, USA) and extracted from the gel matrix using 5% formic acid in 50–100% acetonitrile. Extracted proteins were dried in a SpeedVac vacuum system (Eppendorf Vacufuge Concentrator Model 5301 from Brinkmann, Hamburg, Germany) and stored at −20 °C until LC-MS/MS analyses.

Digested proteins were resuspended in 1% formic acid for chromatographic separation, and eluted peptides were subject to electrospray ionization for MS. A nanoAcquity liquid chromatographer (Waters, Milford, MA, USA) coupled to an LTQ-Orbitrap Velos (ThermoFisher Scientific, Waltham, MA, USA) mass spectrometer was used. The top 15 most abundant peptides (minimum intensity of 500 counts) from each MS scan were selected and fragmented in the linear ion trap. Data were acquired using the Thermo Xcalibur v2.2 software (ThermoFisher Scientific, Waltham, MA, USA).

Collected data were used to search against a modified version of the public database Uniprot containing selected IUIS protein entries using Thermo Proteome Discoverer (v.1.4.1.14) software (ThermoFisher Scientific, Waltham, MA, USA) and Mascot as the search engine. Results were filtered to include proteins identified with at least two medium-confidence peptides (FDR ≤ 5%).

### 5.6. Sequence Identity

These analyses included Uniprot sequences from all isoforms of wasp allergen groups 1, 5, 2 and 3 from *Vespula* spp. and *Polistes* spp. described in https://www.allergen.org/ and https://www.allergome.org/, with reported allergenic capacity. All allergens with complete sequences were used and analyzed using Clustal Omega tool from Services of the EMBL’s European Bionformatics Institute (https://www.ebi.ac.uk/jdispatcher/msa/clustalo, accessed on 14 November 2022) to compare sequence identity. The sequences compared are included in Table S1.

### 5.7. In Silico Structure Prediction

Sequences from group 1 and group 5 major allergens in *P. dominula* and *V. vulgaris*, including Pol d 1 (accession code Q6Q252), Ves v 1 (P49369), and Pol d 5 (P81656), were retrieved from the UniProt database, and their 3D structures were predicted using the AlphaFold Protein structure Database (https://alphafold.ebi.ac.uk/) from the European Bioinformatics Institute (EBI) [32,33]. We also used the available crystallographic structure of Ves v 5 [34] (Protein Data Bank reference 1qnx, https://www.rcsb.org), which is more reliable than in silico structure prediction, to compare the structures within each group (Pol d 1 vs. Ves v 1 and Pol d 5 vs. Ves v 5).

3D alignment was performed using the Pairwise Structure Alignment from RCSB Protein Data Bank (https://www.rcsb.org/) [35]. The Uniprot sequences used were: Q6Q252 (Pol d 1.0101), P49369 (Ves v 1.0101), P81656 (Pol d 5.0101) and Q05110 (Ves v 5.0101).

## Figures and Tables

**Table 1:** Results from LC/MS analyses and isoforms identified. Sequences obtained from IUIS webpage (https://www.allergen.org/ or https://www.allergome.org/).

	Sequence	Isoform	Score	Coverage (%)	No. Peptides	No. Unique Peptides
*Polistes dominula* sequences	XP_015174445.1	Pol d 3	1495.2	47.10	30	30
	Q6Q250	Pol d 1.0103	1661.9	68.67	26	0
	Q6Q249	Pol d 1.0104	1635.1	68.67	25	2
	Q6Q252	Pol d 1.0101	1524.2	64.39	26	0
	Q6Q251	Pol d 1.0102	1512.4	68.67	24	0
	Q7Z269	Pol d 4.0101	2110.7	70.40	15	15
	P81656	Pol d 5.0101	917.36	77.97	16	2
*Vespula* spp. sequences	B1A4F7	Ves v 3.0101	913.9	21.52	14	14
	P49370	Ves v 2.0101	1600.7	41.99	18	0
	Q5D7H4	Ves v 2.0201	2423.8	65.88	23	2
	Q05FZ1	Ves g 2	2374.3	63.78	21	0
	Q05FZ2	Ves g 2	1622.2	49.24	19	1
	P0CH86	Ves s 1.0101	5119.5	65.77	22	21
	Q3ZU95	Ves g 1	3801.2	84.33	18	6
	P49369	Ves v 1.0101	2108.8	60.42	16	2
	P51528	Ves m 1.0101	1917.8	57.00	13	1
	Q05110	Ves v 5.0101	3501.8	75.33	20	1
	P35785	Ves p 5.0101	3011.2	87.25	20	6
	P35784	Ves g 5.0101	2775.3	70.59	20	5
	P35760	Ves m 5.0101	2543.1	74.51	20	5
	P35786	Ves s 5.0101	2469.9	80.00	17	13

## Data Availability

The original contributions presented in this study are included in the article/Supplementary Materials. Further inquiries can be directed to the corresponding author.

## Supplementary Materials

The following supporting information can be downloaded at: https://www.mdpi.com/article/10.3390/toxins18040190/s1, Figure S1: Alignment of the indicated isoform sequences of group 1 allergens from *Polistes* and *Vespula*; Figure S2: Phylogenetic tree of sequences of group 1 allergens from *Polistes* and *Vespula*; Figure S3: Alignment of the indicated isoform sequences of group 5 allergens from *Polistes* and *Vespula*; Figure S4: Phylogenetic tree of sequences of group 5 allergens from *Polistes* and *Vespula*; Table S1: Sequences and corresponding allergen isoforms retrieved from UniProt for structural and homology modeling.

## Abbreviations

The following abbreviations are used in this manuscript:

SDS-PAGE	Sodium dodecyl sulfate–polyacrylamide gel electrophoresis
LC-MS/MS	Liquid Chromatography–Mass Spectrometry tandem analysis
VIT	Venom immunotherapy
CHMP	Committee for Medicinal Products for Human Use
EMA	European Medicines Agency
