# Supplementary material for: Homology Analysis of Polistes dominula and Vespula spp. Venoms: A Comparative In Vitro and In Silico Study
Source: Toxins (Basel). 2026 Apr 18;18(4):190. doi: 10.3390/toxins18040190 (PMC13120226; doi:10.3390/toxins18040190)
Supplement: Supplementary file 1 [file toxins-18-00190-s001.zip › toxins-4185702-supplementary.pdf]

Supplementary Materials Homology Analysis of *Polistes dominula* and *Vespula* spp. Venoms: A Comparative *in vitro* and *in silico* Study

María Morales [1]*, Alicia Jordá Marín [1], Bárbara Cases [1], Louise Wallace [2], Dolores Hernández Fernández De Rojas [1]

Supplementary Tables

Table S1. Sequences and corresponding allergen isoforms retrieved from UniProt for structural and homology modelling.

	***Vespula* spp.**		***Polistes* spp.**	
	**Sequence ID**	**Allergen isoform**	**Sequence ID**	**Allergen isoform**
**Group 1 Phospholipase**	Q3ZU95	Ves g 1	Q9U6W0	Pol a 1.0101
	P51528	Ves m 1.0101	Q6Q249	Pol d 1.0104
	P49369	Ves v 1.0101	Q6Q250	Pol d 1.0103
	P0CH86	Ves s 1.0101	Q6Q252	Pol d 1.0101
			Q6Q251	Pol d 1.0102
**Group 5**	P35785	Ves p 5.0101	P81656	Pol d 5.0101
	P35784	Ves g 5.0101	P83377	Pol g 5.0101
	P35783	Ves f 5.0101	P35780	Pol f 5.0101
	Q05110	Ves v 5.0101	Q05109	Pol a 5.0101
	P35760	Ves m 5.0101	P35759	Pol e 5.0101
	P35786	Ves s 5.0101		
	P35787	Ves vi 5.0101		
**Group 2**	Q05FZ1	Ves g 2	XP_015179722.1	Pol d 2
	Q5D7H4	Ves v 2.0101		
	P493370	Ves v 2.0101		
	Q05FZ2	Ves g 2		
**Group 3**	B1A4F7	Ves v 3.0101	XP_015174445	Pol d 3

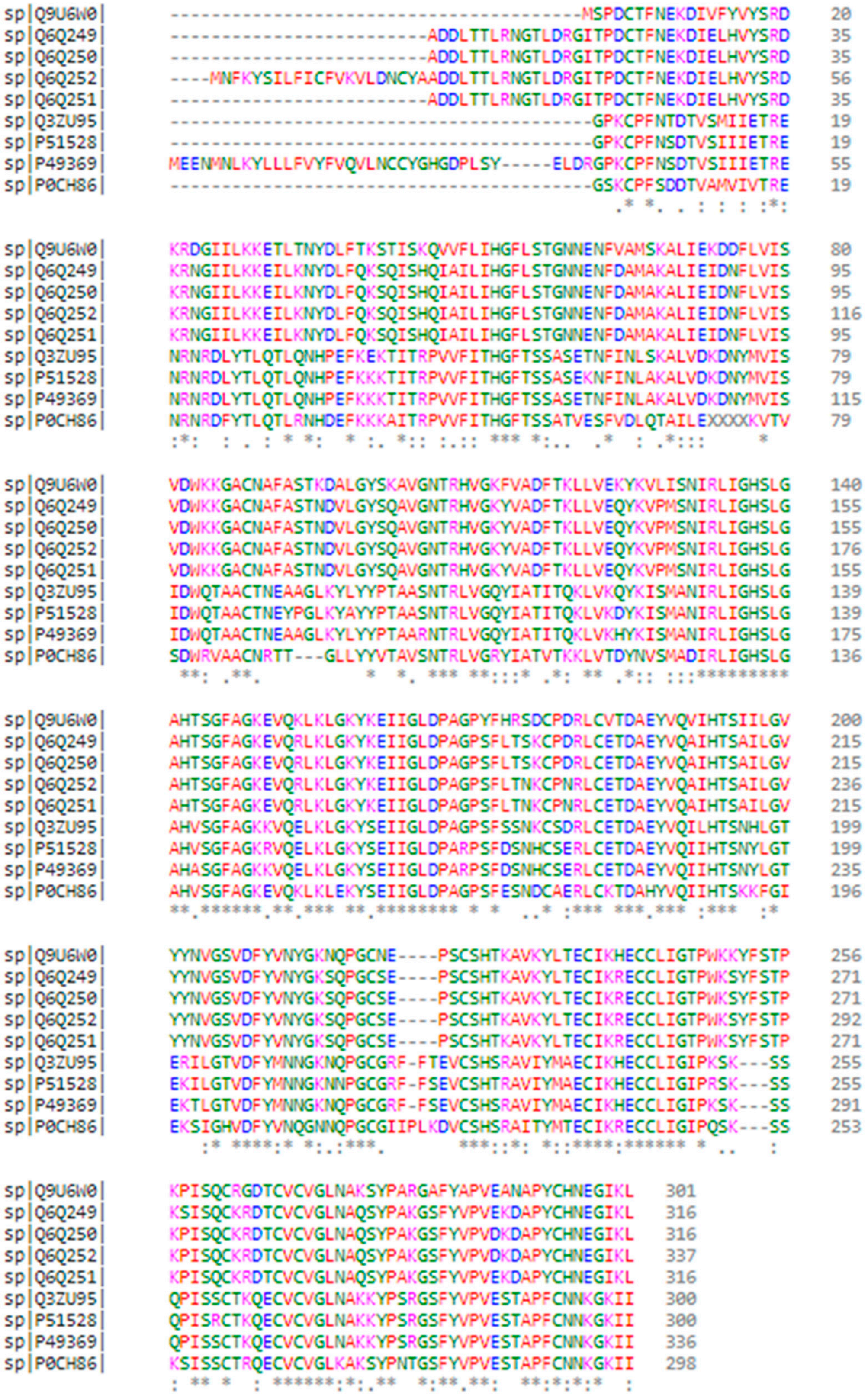

Supplementary Figures

Figure S1. Alignment of the indicated isoform sequences of group 1 allergens from *Polistes* and *Vespula*.

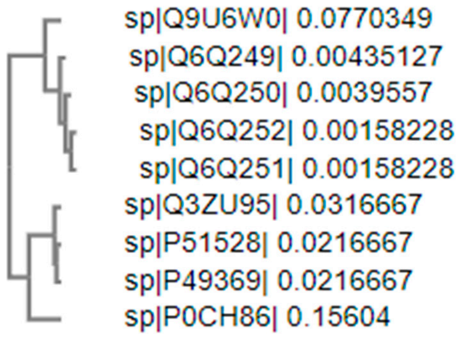

Figure S2. Phylogenetic tree of sequences of group 1 allergens from *Polistes* and *Vespula*.

```
sp|P81656|      MKISCLICLVIVLTIIHLS--QANDYCKIKCS-SGVHTVCQYGESTKPSKNCAGKLIKSV     57
sp|P83377|      -----------------------NDYCKIKCS-SGVHTVCQYGESTKPSKNCAGKVIKSV     36
sp|P35780|      -----------------------VDYCKIKCS-SGIHTVCQYGESTKPSKNCADKVIKSV     36
sp|Q05109|      -----------------SS--QGVDYCKIKCP-SGIHTVCQYGESTKPSKNCAGKVIKSV     40
sp|P35759|      MEIGGLVYLVIVVAIIHSS--QGVDYCKIRCP-SGIHTVCQYGESTKPSKNCAGKVIKSV     57
sp|P35785|      -----------------------NNYCKIKCLKGGVHTACKYGS-LKP--NCGNKIVVSY     34
sp|P35784|      -----------------------NNYCKIKCLKGGVHTACKYES-LKP--NCANKKVVAY     34
sp|P35783|      -----------------------NNYCKIKCLKGGVHTACKYGS-LKP--NCGNKVVVSY     34
sp|Q05110|      MEISGLVYLIIIVTIIDLPYGKANNYCKIKCLKGGVHTACKYGS-LKP--NCGNKVVVSY     57
sp|P35760|      -----------------------NNYCKIKCLKGGVHTACKYGS-LKP--NCGNKKVVSY     34
sp|P35786|      -----------------------VDYCKIKCLKGGVHTACKYGTSTKP--NCGNMVVKSY     35
sp|P35787|      ---------------------KVNYCKIKCLKGGVHTACKYGTSTKP--NCGKMVVKAY     36
                                        :****:*  .*:**.*:*    **  **.   : :

sp|P81656|      GPTEEEKKLIVEEHNRFRQKVAKGLETRGNPGPQPAASNMNNLVWNDELAKIAQVWASQC     117
sp|P83377|      GPTEEEKKLIVEEHNRFRQKVAQGLETRGNPGPQPAASNMNNLVWNDEQAKIAQVWASQC     96
sp|P35780|      GPTEEEKKLIVNEHNRFRQKVAQGLETRGNPGPQPAASDMNNLVWNDELAHIAQVWASQC     96
sp|Q05109|      GPTEEEKKLIVSEHNRFRQKVAQGLETRGNPGPQPAASDMNDLVWNDELAHIAQVWASQC     100
sp|P35759|      GPTEEEKKLIVSEHNRFRQKVAQGLETRGNPGPQPAASDMNDLVWNDELAHIAQVWASQC     117
sp|P35785|      GLTKEEKQDILKEHNDFRQKIARGLETRGNPGPQPPAKNMKNLVWNDELAYVAQVWANQC     94
sp|P35784|      GLTKQEKQDILKEHNDFRQKIARGLETRGNPGPQPPAKNMKNLVWSDELAYIAQVWANQC     94
sp|P35783|      GLTKQEKQDILKEHNDFRQKIARGLETRGNPGPQPPAKNMKNLVWNDELAYVAQVWANQC     94
sp|Q05110|      GLTKQEKQDILKEHNDFRQKIARGLETRGNPGPQPPAKNMKNLVWNDELAYVAQVWANQC     117
sp|P35760|      GLTKQEKQDILKEHNDFRQKIARGLETRGNPGPQPPAKNMKNLVWSDELAYIAQVWANQC     94
sp|P35786|      GVTQAEKQEILKIHNDFRNKVARGLETRGNPGPQPPAKNMNNLVWNNELANIAQIWASQC     95
sp|P35787|      GLTEAEKQEILKVHNDFRQKVAKGLETRGNPGPQPPAKNMNNLVWNDELANIAQVWASQC     96
                * *: **: *:. ** **:*:*:************ *.:*::***.:* * :**:**.**

sp|P81656|      QILVHDKCRNTEKYQVGQNIAYAGSS-NHFPSVTKLIQLWENEVKDFNYNTGITNKNFGK     176
sp|P83377|      QILVHDKCRNTEKYQVGQNIAYAGSS-NHFPSVTKLIQLWENEVKDFNYNTGITNKNFGK     155
sp|P35780|      QILVHDKCRNTAKYQVGQNIAYAGGS--KLPDVVSLIKLWENEVKDFNYNKGITKQNFGK     154
sp|Q05109|      QFLVHDKCRNTAKYPVGQNIAYAGGS--NLPDVVSLIKLWENEVKDFNYNTGITKQNFAK     158
sp|P35759|      QFLVHDKCRNTAKYPVGQNIAYAGGS--KLPDVVSLIKLWENEVKDFNYNTGITKQNFAK     175
sp|P35785|      QY-GHDTCRDVAKYPVGQNVALTGSTADKYDNPVKLVKMWEDEVKDYNPKKKFSENNFNK     153
sp|P35784|      QY-GHDTCRDVAKYPVGQNVALTGSTAAKYDNPVKLVKMWEDEVKDYNPKKKFSENNFLK     153
sp|P35783|      QY-GHDTCRDIAKYQVGQNVALTGSTAAKYDDPVKLVKMWEDEVKDYNPKKKFSGNNFLK     153
sp|Q05110|      QY-GHDTCRDVAKYQVGQNVALTGSTAAKYDDPVKLVKMWEDEVKDYNPKKKFSGNDFLK     176
sp|P35760|      QY-GHDTCRDVAKYQVGQNVALTGSTAAVYNDPVKLVKMWEDEVKDYNPKKKFSENNFLK     153
sp|P35786|      KY-GHDTCKDTTKYNVGQNIAVSSSTAAVYENVGNLVKAWENEVKDFNPTISWEQNEFKK     154
sp|P35787|      NY-GHDTCKDTEKYPVGQNIAKRSTTAALFDSPGKLVKMWENEVKDFNPNIEWSKNNLKK     155
                :   **.*::  ** ****:*  . :     .  .*:: **:****:* .     ::: *

sp|P81656|      VGHYTQMVWGNTKEVGCGSLKYVEKNMQIHYLICNYGPAGNYLGQPIYTKK     227
sp|P83377|      VGHYTQMVWGNTKEVGCGSLKYVEKNMKIHYLICNYGPAGNYLGQPIYTKK     206
sp|P35780|      VGHYTQMIWAKTKEIGCGSLKYMKNNMQHHYLICNYGPAGNYLGQLPYTKK     205
sp|Q05109|      IGHYTQMVWGKTKEIGCGSLKYMENNMQNHYLICNYGPAGNYLGQLPYTKK     209
sp|P35759|      IGHYTQMVWGKTKEIGCGSLKYMENKMQNHYLICNYGPAGNYLGQLPNTK-     225
sp|P35785|      IGHYTQMVWANTKEIGCGSIKYIQNEWHKHYLVCNYGPSGNFGNEELYQTK     204
sp|P35784|      IGHYTQMVWANTKEVGCGSIKYIQDKWHKHYLVCNYGPSGNFGNEELYQTK     204
sp|P35783|      TGHYTQMVWANTKEVGCGSIKFIQEKWHKHYLVCNYGPSGNFQNEELYQTK     204
sp|Q05110|      TGHYTQMVWANTKEVGCGSIKYIQEKWHKHYLVCNYGPSGNFMNEELYQTK     227
sp|P35760|      IGHYTQMVWANTKEVGCGSIKYIQENWHKHYLVCNYGPSGNFQNEELYQTK     204
sp|P35786|      IGHYTQMVWAKTKEIGCGSIKYVDNNWYTHYLVCNYGPAGNFGNQEVYERK     205
sp|P35787|      TGHYTQMVWAKTKEIGCGSVKYVKDEWYTHYLVCNYGPSGNFRNEKLYEKK     206
                 ******:*.:***:****:*::..:   ***:*****:**:  .:
```

Figure S3. Alignment of the indicated isoform sequences of group 5 allergens from *Polistes* and *Vespula*.

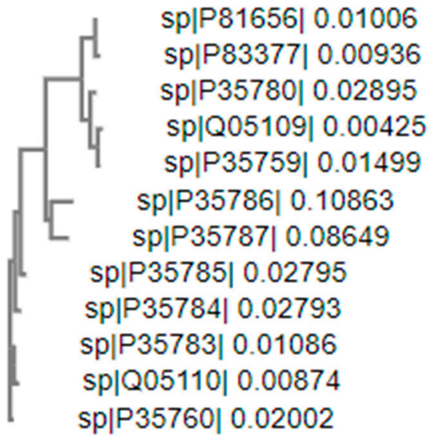

Figure S4. Phylogenetic tree of sequences of group 5 allergens from *Polistes* and *Vespula*.
